# The association of state per capita income and military service deaths in the Vietnam and Iraq wars

**DOI:** 10.1186/1478-7954-7-1

**Published:** 2009-01-06

**Authors:** Charles Maynard

**Affiliations:** 1 Department of Health Services, University of Washington, Seattle, Washington, USA

## Abstract

**Background:**

In the United States, social burdens including war casualties are often distributed unequally across groups of individuals, communities, and states. The purpose of this report was to examine the association between war deaths and per capita income in the 50 states and District of Columbia during the Vietnam and Iraq wars.

**Methods:**

The numbers of deaths by the home state of record for each conflict were obtained from Department of Defense records on the Internet as were key variables including age at death, gender, race, branch of service, rank, circumstances of death, home state of record and the ratio of wounded to dead. In addition, we obtained state per capita income and state population for the relevant times.

**Results:**

Characteristics of decedents in the 2 conflicts were very similar with young, white enlisted men accounting for the majority of deaths. However, in the Iraq war, women accounted for a 2.4% of casualties. Also of note was the higher ratio of wounded to dead in Iraq. At the level of the state, the correlation between the ratio of deaths per 100,000 and per capita income was -0.51 (p < 0.0001) for Vietnam and -0.52 for Iraq (p < 0.0001). In both eras, states with lower per capita income tended to have higher ratios of deaths per population.

**Conclusion:**

For military service members serving in the Vietnam and Iraq conflicts, there were many more women who died in the latter war. Whether war deaths resulted in lower per capita income cannot be determined from these cross sectional data; we simply note a strong association between per capita income and war casualty rates for both wars.

## Background

In the United States, social burdens including war casualties are often distributed unequally across groups of individuals, communities, and states. In both Vietnam and Iraq the majority of deaths occurred in white enlisted men who served in the US Army or Marine Corps in hostile situations [1-4]. Less is known about the association between war casualties and income of states. The purpose of this report was to examine the association between war deaths and per capita income in the 50 states and District of Columbia during the Vietnam and Iraq wars. A secondary objective was to compare the characteristics of individuals who died in the 2 conflicts.

## Methods

The numbers of deaths by the home state of record for each conflict were obtained from the World Wide Web [2,4] as were key variables including age at death, gender, race, branch of service, rank (enlisted versus officer or warrant officer), circumstances of death (hostile versus non-hostile), and the home state of record. Hostile deaths refer to those personnel who were killed in action, died of wounds, were missing in action, or died in captivity. Tabular counts of decedents (e.g., number of deaths by age) for the Vietnam war were obtained from the National Archives of the United States, which in turn received archival records from the Department of Defense [2]. Detailed individual level characteristics of decedents in the Iraq war were obtained from publicly available files provided by the Defense Manpower Data Center [4]. Death information for the Iraq war is changing as the war is ongoing. The ratio of wounded to dead was calculated for the conflicts in Vietnam and Iraq [5,6]. Race was defined differently in the 2 periods; in order to compare racial characteristics during the 2 time periods, race was categorized as white or non-white.

The ratio of state deaths to state population was calculated as the number of deaths per state of record to state population in either 1970 or 2000. State population figures for the years 1970 and 2000 were obtained from the Pennsylvania State Data Center [7]. Per capita income in 1999 dollars for the years 1969 and 1999 (the latest year for which information was available) was obtained from the US Census [8]. The association between per capita income and the ratio of the number of deaths to state population was examined with the Spearman rank correlation coefficient for the Vietnam and Iraq wars separately.

## Results

The numbers of deaths in Vietnam exceeded those in Iraq by more than 10 times as deaths in Vietnam spanned the years 1956 through 1998, whereas those for the ongoing Iraq war covered the years 2003 to July 2008. Vietnam deaths after 1975 were for individuals whose status changed from missing to deceased. The characteristics of casualties in Vietnam and Iraq were similar, with the exception that the number of women increased significantly in Iraq (table 1). In Vietnam, there were only 8 recorded deaths in women, whereas in the Iraq there were 92 women who died. Another difference was that the ratio of wounded to dead was much higher in Iraq.

**Table 1:** Characteristics of casualties in the Vietnam and Iraq wars

**Characteristic**	**Vietnam** **(n = 58,193)**	**Iraq** **(n = 4106)**
Age in years (median)	21	24
Men	100%	98%
White	86%	75%
United States Army	66%	72%
United States Marine Corps	25%	24%
Enlisted personnel	86%	91%
Hostile death	81%	81%
Ratio of wounded to dead	2.6/1	7.5/1

**Figure 1:**
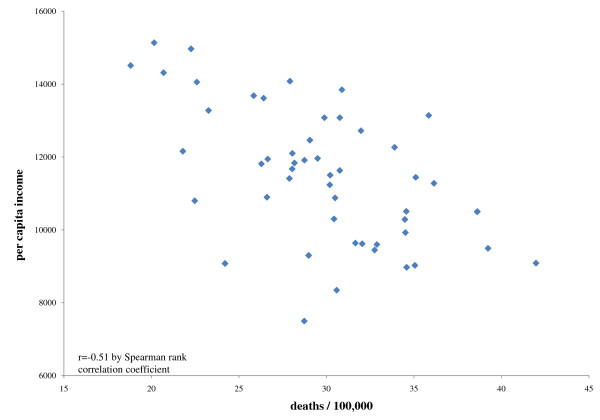
**Combat casualties and state per capita income during the Vietnam war**.

**Figure 2:**
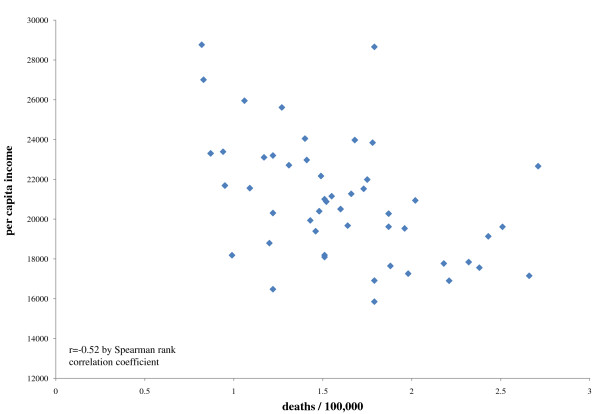
**Combat casualties and state per capita income during the Iraq war**.

As seen in the table 2, the average per capita income in 1969 in 1999 dollars was $11.5 thousand compared to $20.9 thousand in 1999. The ratio of deaths per 100,000 was 29.8 for Vietnam and 1.6 for Iraq. The correlation between the ratio of deaths per 100,000 and per capita income was -0.51 (p < 0.0001) for Vietnam and -0.52 for Iraq (p < 0.0001). In both eras, states with lower per capita income tended to have higher ratios of deaths per population (figures 1 and 2).

**Table 2:** Ratio of deaths to population and per capita income by state during the Vietnam and Iraq wars

State	# deaths/100,000 (Vietnam)	# deaths/100,000 (Iraq)	Per capita income (1969)	Per capita income (1999)
Alabama	35.05	1.51	9,026	18,189
Alaska	18.81	2.71	14,511	22,660
Arizona	35.10	1.87	11,442	20,275
Arkansas	30.58	2.21	8,345	16,904
California	27.91	1.31	14,079	22,711
Colorado	28.05	1.40	12,100	24,049
Connecticut	20.15	0.82	15,135	28,766
Delaware	31.97	0.87	12,719	23,305
District of Columbia	22.26	1.79	14,967	28,659
Florida	28.74	1.09	11,913	21,557
Georgia	34.48	1.55	10,285	21,154
Hawaii	35.84	1.73	13,140	21,525
Idaho	30.43	2.32	10,300	17,841
Illinois	26.41	1.17	13,615	23,104
Indiana	29.49	1.48	11,960	20,397
Iowa	30.19	1.64	11,235	19,674
Kansas	27.88	1.60	11,410	20,506
Kentucky	32.75	1.51	9,447	18,093
Louisiana	24.20	1.79	9,077	16,912
Maine	34.51	1.96	9,926	19,533
Maryland	25.84	1.27	13,682	25,614
Massachusetts	23.26	1.06	13,276	25,952
Michigan	29.88	1.49	13,078	22,168
Minnesota	28.17	1.22	11,835	23,198
Mississippi	28.73	1.79	7,499	15,853
Missouri	30.21	1.43	11,500	19,936
Montana	38.62	2.66	10,503	17,151
Nebraska	26.60	2.51	10,896	19,613
Nevada	30.88	1.75	13,845	21,989
New Hampshire	30.76	1.78	11,629	23,844
New Jersey	20.69	0.83	14,313	27,006
New Mexico	39.23	1.98	9,494	17,261
New York	22.59	0.94	14,056	23,389
North Carolina	31.65	1.22	9,638	20,307
North Dakota	32.04	2.18	9,618	17,769
Ohio	29.05	1.51	12,462	21,003
Oklahoma	38.61	1.88	10,495	17,646
Oregon	33.89	2.02	12,264	20,940
Pennsylvania	26.64	1.52	11,944	20,880
Rhode Island	21.79	0.95	12,158	21,688
South Carolina	34.58	1.20	8,972	18,795
South Dakota	28.98	2.38	9,299	17,562
Tennessee	32.88	1.46	9,599	19,393
Texas	30.49	1.87	10,877	19,617
Utah	34.56	0.99	10,507	18,185
Vermont	22.47	3.45	10,799	20,625
Virginia	28.04	1.68	11,671	23,975
Washington	30.76	1.41	13,078	22,973
West Virginia	41.97	1.22	9,089	16,477
Wisconsin	26.28	1.66	11,812	21,271
Wyoming	36.14	2.43	11,278	19,134

## Discussion

Much has been said about similarities and differences between the conflicts in Vietnam and Iraq. The former was fought by draftees and volunteers, whereas the current conflict includes only volunteers, as at this time there is no military draft in the United States. One striking difference was the numbers of deaths in Vietnam far exceeded those in the ongoing Iraq war. On the other hand, characteristics of decedents in the 2 conflicts were very similar with young, white enlisted men accounting for the majority of deaths. However, in the Iraq war, women accounted for a higher proportion of casualties. Also of note was the difference in the ratio of wounded to dead in the 2 conflicts. This may be an indication of improved medical care in the Iraq war, as personnel with severe injuries are surviving wounds that in previous wars were lethal [9].

It was also the case that states with fewer resources as measured by per capita income experienced higher casualty rates in both conflicts. The magnitude of the association was relatively strong and remarkably similar in both eras. For states and the armed forces, this meant the loss of young men and women; the loss of young women is particularly applicable to the Iraq conflict, where women currently account for 2.4% of deaths [2,4].

A major limitation of the study was that the state population was used as the denominator. A better denominator would have been the number of combatants from each state, as it is likely that individuals from poorer states were more likely to join the military than were those from wealthier states. Unfortunately, it was not possible to obtain counts of the number of combatants by state.

While there could have been selection bias in that men and women from poorer states were more likely to join the military, this does not change the fact that poorer states had higher casualty rates. This study was based on publicly available data over which the author had little control; information was used as provided. For example, 1999 was latest year for which state per capita income was reported; this was 4 years prior to the start of the Iraq War. Furthermore, the information regarding deceased combatant's characteristics was taken "as is." The author was not able to assess the accuracy of the information; this applies particularly to the Vietnam war which started over 45 years ago.

## Conclusion

In the Vietnam and Iraq conflicts, the burden of war casualties was shouldered primarily by young enlisted men, but as seen in Iraq, young women are beginning to share this burden. Less appreciated is the fact that populations may be affected by war casualties in military personnel. Whether war deaths cause states to have lower income cannot be determined from these cross sectional data; we simply note a strong association between per capita income and war casualty rates in the 2 conflicts.

## Competing interests

The author declares that they have no competing interests.

## Authors' contributions

Charles Maynard is the sole author of this study and was responsible for study design, conception, data analysis and interpretation. He also drafted the manuscript, critically revised it for important intellectual content, and approved the final version of manuscript.
